# Effects of adding various salted seafood (*jeotgal*) types on the microbial, metabolic, and sensory dynamics of kimchi during fermentation

**DOI:** 10.1016/j.fochx.2026.103832

**Published:** 2026-04-05

**Authors:** Ju Young Lim, Yun-Jeong Choi, Ji-Young Choi, Sung Hee Park, Sung Gi Min, Mi-Ai Lee

**Affiliations:** a Kimchi Factory Research Group, World Institute of Kimchi, Gwangju 61755, Republic of Korea; b Sustainable Distribution Research Group, World Institute of Kimchi, Gwangju 61755, Republic of Korea; c Department of Food and Nutrition, Kyung Hee University, Seoul 02447, Republic of Korea

**Keywords:** Kimchi quality, Salted seafood, Metabolites, Microbial community, Fermentation

## Abstract

Kimchi is a traditional Korean fermented dish in which salted seafood is widely used; however, its specific role in fermentation remains unclear. This study investigated the impact of four salted seafoods (anchovy, cutlass offal, yellow croaker, shrimp) on the physicochemical, microbial, metabolic, and sensory properties of kimchi compared to a no-seafood control. Samples were fermented at 4 °C for 6 weeks. Salted seafood accelerated acidification and increased total and amino nitrogen, hypoxanthine, inosine, and free amino acids (e.g., glutamic acid, γ-aminobutyric acid). Halophilic lactic acid bacteria, including *Tetragenococcus halophilus*, were enriched in anchovy kimchi at early stages, whereas *Latilactobacillus sakei* dominated all samples at mid–late fermentation. Metabolomic profiling and electronic tongue analysis revealed enhanced accumulation of flavor-related metabolites and stronger umami and sourness in seafood-supplemented kimchi, associated with organic acids and microbial succession. Overall, salted seafood functions as both a flavor enhancer and natural microbial starter in kimchi fermentation.

## Highlights

• Salted seafood accelerated fermentation and increased amino nitrogen in kimchi.
• Microbial succession varied by seafood type, stabilizing kimchi fermentation.
• Salted seafood served as a microbial starter influencing fermentation.
• Sensory changes were associated with microbial shifts in seafood-supplemented kimchi.
• Bioactive and flavor compounds increased in salted seafood-supplemented kimchi.

## 1 Introduction

Kimchi is a traditional Korean fermented food, typically prepared using cabbage or radish combined with seasonings, such as red pepper powder, garlic, ginger, and salted seafood (Kim & Kim, 1994). Among these ingredients, salted seafood is especially important owing to its high protein and amino acid content, which enhances the umami flavor and supplements the low protein levels of the main vegetable components (Liu & Ralston, 2021). Salted seafood contains a variety of endogenous enzymes that remain active even under high-salt conditions, supporting fermentation and enhancing amino acid production (Jiang et al., 1991; Oh et al., 1997; Yongsawatdigul et al., 2007). In addition, halophilic bacteria present in salted seafood contribute to the production of organic acids and metabolites through their metabolic activity (Shim et al., 2022). These compounds, including free amino acids, impart umami and unique flavor characteristics, improving the taste quality of kimchi upon the addition of salted seafood (Koo et al., 2016).

Beyond kimchi, many traditional fermented foods, such as fish sauce and soy sauce, rely on raw materials that serve simultaneously as substrates and sources of indigenous microbiota and enzymatic activities (Feng et al., 2023; Han, Hu, et al., 2024). This phenomenon is closely related to the broader concept of terroir, which describes how the characteristics of an agricultural or fermented product arise from the unique ecosystem of its geographic origin, shaped by climate, terrain, and traditional practices (Capozzi et al., 2015; Lee et al., 2023). Differences in ingredient origin and production methods within these ecosystems can lead to distinct fermentation trajectories and region-specific product characteristics. From this perspective, selecting different types of salted seafood (*jeotgal*) represents an important “microbial terroir” that may influence fermentation dynamics and flavor development in kimchi.

In Korea, the term “*jeotgal*” refers broadly to various types of salted and fermented seafood. Commonly consumed varieties include salted anchovies (*myeolchijeot*; *Engraulis japonicus*), hairtail offal (*galchisokjeot*; *Trichiurus lepturus*), yellow croaker (*hwangseogeojeot*; *Collichthys lucidus*), and shrimp (*Saeujoet*; *Acetes japonicus*) (Koo et al., 2016). Previous studies have provided valuable insights into the role of *jeotgal* in shaping microbial dynamics and metabolite profiles in kimchi (Jung et al., 2018; Ko et al., 2004). However, most of these studies are limited to kimchi prepared with only one or two types of salted seafood and examined either microbial succession or metabolic shifts in isolation, with only partial or indirect consideration of sensory attributes (Baek et al., 2023; Choi et al., 2018; Kang et al., 2016). Consequently, it remains unclear how different types of *jeotgal* differentially shape microbial communities, flavor-related metabolites, and taste profiles under identical fermentation conditions, and which *jeotgal* components*,* such as indigenous microorganisms, metabolites, or nitrogen-rich substrates, are primarily responsible for these differences.

The present study aimed to elucidate, in a multi-omics framework, how different salted seafood types influence microbial community dynamics and flavor development in fermented kimchi. We prepared kimchi with four representative *jeotgal*—salted anchovy, hairtail offal, yellow croaker, and shrimp—and compared them with a control without salted seafood. We monitored changes in physicochemical parameters, microbiological properties, metabolite profiles, and sensory characteristics (electronic tongue) during fermentation at 4 °C. By integrating these datasets, we revealed ingredient-specific fermentation trajectories associated with each *jeotgal* type, linked key lactic acid bacteria with amino acids, nucleotides, and other flavor-related metabolites, and evaluated the impact of omitting *jeotgal* on fermentation stability and taste quality. This comprehensive approach transcends simple comparisons of *jeotgal* presence versus absence and highlights salted seafood as a natural microbial and enzymatic starter module that can be rationally selected to optimize kimchi fermentation and flavor.

## 2 Materials and methods

### 2.1 Experimental materials

Salted seafood was prepared and used as described in our previous studies (Lim et al., 2023; Lim et al., 2024). Other ingredients used for kimchi preparation were purchased from the Western Agricultural and Fishery Market in Gwangju, Republic of Korea. High-purity reagents of first-grade quality supplied by Daejung (Gyeonggi-do, Republic of Korea) were used in all analytical procedures. High-pressure liquid chromatography (HPLC)-grade water and acetonitrile (Merck & Co., Inc., Rahway, NJ, USA) were used for chromatographic analyses.

### 2.2 Kimchi preparation

To prepare the kimchi cabbage, it was immersed in a 10% (*w*/*v*) salt solution for 18 h, followed by three rinses with water and a 2-h draining period. After draining, the cabbages were cut into uniform 3 × 3 cm pieces for kimchi preparation. The seasoning mixture comprised 16.7% garlic, 2.7% ginger, 23.3% red pepper powder, and 26.7% salted seafood. For control samples without salted seafood, an equivalent volume of brine was added to ensure a comparable final salinity. The seasoning was combined with the salted kimchi cabbage at a mixing ratio of 85:15 (salted kimchi cabbage: seasoning).

The resultant test groups were as follows: control kimchi without salted seafood (CK), kimchi with salted anchovy (KSA), kimchi with salted hairtail offal (KSH), kimchi with salted yellow croaker (KSC), and kimchi with salted shrimp (KSS). Samples weighing 600 g were vacuum-sealed in polyethylene bags with a vacuum packing device (AZC-070; INTRISE, Ansan, Republic of Korea) and stored in a refrigerator at 4 °C for 6 weeks. Samples were collected at regular 1-week intervals to analyze their characteristics.

### 2.3 Analysis of pH and titratable acidity

To analyze pH and titratable acidity, each kimchi sample was homogenized, and the resulting extract was used for analysis. Analyses were performed at 25 °C with a TitroLine 5000 (SI Analytics GmbH, Mainz, Germany). The sample was titrated with 0.1 M sodium hydroxide (NaOH) until the pH reached 8.3, and the acidity was expressed as lactic acid equivalent (% *w*/w).

### 2.4 Analyses of nitrogen-related factors

#### 2.4.1 Total nitrogen (TN)

TN content was analyzed using the persulfate digestion method following the HACH Method 10,072. A total of 0.5 mL of the sample was mixed with a Total Nitrogen Persulfate Reagent (26718–46; HACH, Loveland, CO, USA) into a Total Nitrogen Hydroxide Digestion Reagent Vial (27140–45; HACH). The mixture was digested at 105 °C for 30 min. After cooling, Total Nitrogen Reagent A (26719–46; HACH) and Total Nitrogen Reagent B (26720–46; HACH) were sequentially added to the vial to initiate the reaction. Subsequently, 2 mL of the reacted mixture was transferred into a Total Nitrogen Reagent C Vial (26721–45; HACH), thoroughly mixed, and the TN level was quantified using a colorimeter (T-6800; Sinsche Technology Co., Ltd., Shenzhen, China).

#### 2.4.2 Amino nitrogen (AN)

AN content was determined using the formol titration method described by Northrop (1926). A total of 2 g of kimchi was mixed with distilled water to reach a final volume of 100 mL, followed by incubation for 30 min to extract soluble nitrogen compounds. Subsequently, 20 mL of this solution was mixed with 20 mL of neutralized formalin and titrated with 0.1 M NaOH until reaching a pH of 8.3. The AN content was determined according to the following formula (E1):

(E1) $$\mathrm{AN content}\;\left(\mathrm{mg}/100g\right)=\frac{\left(A-B\right)\times 1.4\times 0.1\;N\;\text{NaOH factor}\times D}{\text{sample weight}\;\left(g\right)}\times 1,000\;$$

where A is the volume of 0.1 M NaOH consumed by the sample (mL), B is the volume consumed by the blank (mL), 1.4 is the conversion factor (mg AN per mL NaOH), and D is the dilution factor.

### 2.5 Microbial analysis

Kimchi samples were homogenized using a sterilized blender and aseptically transferred into sterile filter bags. The filtrate was manually extracted and serially diluted in 0.85% sterile saline solution. The diluted samples were then inoculated on Coliform Count plates (Petrifilm™ CC plates; 3 M™, Seoul, Republic of Korea) for coliforms, Aerobic Count plates (Petrifilm™ AC plates) for aerobic bacteria, Lactic Acid Bacteria Count plates (Petrifilm™ LAB plates) for lactic acid bacteria, and Yeast and Mold plates (Petrifilm™ YM plates) for fungal counts. The CC plates were incubated at 35 °C for 24 h, the AC and LAB plates were incubated at 35 °C for 48 h, and the YM plates were incubated at 25 °C for 120 h. Microbial counts were measured in triplicate and expressed as log CFU/g.

### 2.6 Microbial community analysis

Microbial genomic DNA was isolated from kimchi samples using the Maxwell RSC PureFood GMO and Authentication Kit (Promega, Madison, WI, USA), following the manufacturer's protocol. The V3–V4 regions of the bacterial 16S rRNA gene were amplified using fusion primers optimized for microbial community profiling. Polymerase chain reaction was conducted under the following conditions: initial denaturation at 95 °C for 3 min, followed by 25 cycles at 95 °C for 30 s, 55 °C for 30 s, and 72 °C for 30 s, with a final extension at 72 °C for 5 min. Amplified products were verified using 1% agarose gel electrophoresis and visualized using a Gel Doc system (Bio-Rad Laboratories, Hercules, CA, USA). Amplicons were purified using CleanPCR reagents (CleanNA, Waddinxveen, Netherlands), and equal concentrations of the purified DNA were pooled. Non-target fragments were removed during this step. The pooled DNA libraries were assessed for size and quality using a Bioanalyzer 2100 system equipped with a DNA 7500 chip (Agilent Technologies, Santa Clara, CA, USA). Sequencing was performed using the Illumina MiSeq platform (Illumina Inc., San Diego, CA, USA) by CJ Bioscience, Inc. (Seoul, Republic of Korea) following standard protocols.

Raw sequencing reads were subjected to quality control, including removal of low-quality sequences (Q < 25), primer trimming, and dereplication using VSEARCH. Chimeric sequences were identified and excluded using UCHIME, and taxonomic classification was performed using the EzBioCloud 16S rRNA reference database. Operational taxonomic units were clustered, with singleton reads excluded from downstream analysis. Alpha diversity metrics, such as Shannon, Simpson, and ACE, and beta diversity indices, including Bray–Curtis and Jensen–Shannon, were calculated. Functional inference was performed using PICRUSt and MinPath. Biomarker identification was conducted through linear discriminant analysis effect size analysis, and relevant statistical tests. All bioinformatic analyses were executed via the 16S-based MTP cloud platform of EzBioCloud (CJ Bioscience Inc., Seoul, Republic of Korea).

### 2.7 Analyses of free amino acids and nucleic acid-related compounds

To analyze free amino acids and nucleic acid-related compounds, kimchi samples were first homogenized and diluted, and the resulting filtrates were used for chromatographic analysis. HPLC was performed using an Ultimate 3000 (Thermo Dionex; Thermo Fisher Scientific, Waltham, MA USA) equipped with an Inno C18 column (4.6 × 150 mm, 5 μm; Youngjin Biochrom, Gyeonggi-do, Republic of Korea) for amino acid analysis and an Inno C18 column (4.6 × 250 mm, 5 μm; Youngjin Biochrom) for the separation of nucleic acid-related compounds.

For free amino acid analysis, the mobile phases consisted of 40 mM sodium phosphate buffer (mobile phase A) and a mixture of water, acetonitrile, and methanol in a 10:45:45 (*v*/v/v) ratio (mobile phase B). For nucleic acid-related compounds analysis, mobile phase A comprised 0.05 M potassium phosphate buffer, whereas mobile phase B comprised potassium phosphate and methanol in a 90:10 (*v*/v) ratio. The flow rates were set at 1.5 mL/min and 0.7 mL/min for amino acids and nucleic acid-related compounds, respectively. The oven temperature was set at 40 °C.

### 2.8 Electronic tongue (e-tongue) analysis

To evaluate taste attributes, each kimchi sample was diluted 50- to 100-fold with distilled water and filtered prior to measurement. The resulting filtrate was analyzed using an α-Astree II electronic tongue system (Alpha MOS, Toulouse, France) equipped with a sensor array composed of seven chemically modified field-effect transistor sensors. The sensor identifiers, AHS (sourness), ANS (sweetness), CTS (saltiness), NMS (umami), SCS (bitterness), CPS, and PKS (comprehensive taste), denote proprietary sensor IDs. An Ag/AgCl reference electrode completed the measurement setup, and all measurements were conducted in five replicates. The ChemFET sensors are coated with copolymer-based membranes containing chemically selective compounds that generate cross-selective responses. Therefore, taste characteristics are interpreted based on integrated sensor response patterns using multivariate analysis rather than individual sensor–one-taste signals (Lorenz et al., 2009); the performance and sensitivity of this sensor platform have been previously validated (Yang et al., 2013). Voltage–time signals were recorded for 120 s, with the final 30 s used for data analysis.

### 2.9 Metabolomic analysis

For metabolomic profiling, freeze-dried kimchi samples were derivatized before gas chromatography–mass spectrometry (GC–MS) analysis. Samples were first incubated with 100 μL of O-methoxyamine hydrochloride in pyridine solution (20 mg/mL) in the dark at 30 °C for 90 min. Subsequently, 50 μL of *N*-methyl-*N*-trimethylsilyl-trifluoroacetamide containing 1% trimethylchlorosilane was added to initiate silylation. The mixture was vortexed for 30 s and incubated at 37 °C for 30 min. Ribitol (10 μL, 0.5 mg/L) was added as an internal standard. Following centrifugation at 13,000 rpm for 10 min, the supernatant was analyzed using a GC–MS (QP2020, Shimadzu, Kyoto, Japan). Metabolite separation was performed on an Rtx-5MS capillary column (30 m × 0.25 mm ID; J&W Scientific, Rancho Cordova, CA, USA). The inlet temperature was set at 230 °C. The column temperature was initially maintained at 80 °C for 2 min, ramped at 15 °C/min to 330 °C, and held isothermally for an additional 6 min. The transfer line and ion source were maintained at 250 °C and 200 °C, respectively. Electron ionization was performed at 70 eV, with helium serving as the carrier gas at a constant flow rate of 1 mL/min. Spectral data were acquired at a scan rate of 20 scans per second over a mass range of 85–500 *m*/*z* using the Shimadzu GC solution (Shimadzu, Kyoto, Japan). Metabolites were identified by comparing spectra with the AIoutput software, NIST 14.0 library, and the human metabolome database (HMDB, http://www.hmdb.ca).

### 2.10 Statistical analysis

Statistical analyses were performed using GraphPad Prism 10.4.1 (GraphPad Software, Inc., San Diego, CA, USA) and the R statistical system 4.3.3 (R Development Core Team, 2022). Multiple unpaired *t*-tests and one-way analysis of variance tests were conducted for group comparisons, with statistical significance defined as *P* < 0.05. Duncan's multiple range test was performed for post hoc testing to test for significant inter-sample differences (*P* < 0.05). Principal component analysis (PCA) was used to identify major trends and underlying structures in the e-tongue and metabolite data of kimchi. PCA was performed on the metabolite profiles at weeks 0 and 6, with bacterial and electronic tongue data incorporated as supplementary quantitative variables, using the FactoMineR R package. Hierarchical clustering heatmaps were created using the pheatmap R package to visualize the patterns of metabolite distribution. Pearson correlation coefficients were calculated to examine the associations among metabolite profiles, microbial abundance, and sensory attributes, with statistical significance indicated by asterisks (*P* < 0.05).

## 3 Results and discussion

### 3.1 Quality indicator changes of kimchi during fermentation

#### 3.1.1 Changes in physicochemical characteristics

**Fig. 1:**
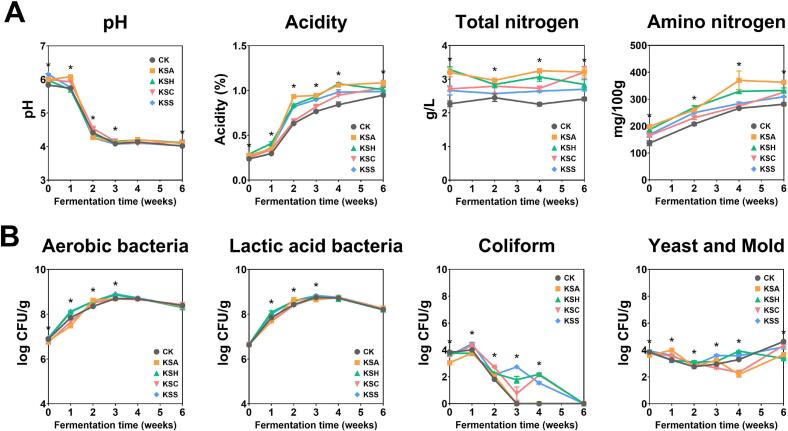
Changes in (A) physicochemical and (B) microbiological characteristics of Kimchi during fermentation. Kimchi samples were prepared with different salted seafood: CK (control; kimchi without salted seafood), KSA (kimchi with salted anchovy), KSH (kimchi with salted hairtail offal), KSC (kimchi with salted yellow croaker), and KSS (kimchi with salted shrimp). Data are presented as mean ± standard deviation. Asterisks (*) denote statistically significant correlations (*p* < 0.05). (For interpretation of the references to colour in this figure legend, the reader is referred to the web version of this article.)

During fermentation, pH and titratable acidity of fermented foods fluctuate dynamically, driven by the microbial production of organic acids, such as lactic acid and citric acid (Park et al., 2023). These parameters serve as key indicators of the quality of fermented products. As depicted in Fig. 1A, all kimchi samples exhibited a sharp decrease in pH and a concurrent increase in titratable acidity during the first two weeks of fermentation, regardless of the addition of salted seafood. The initial pH of all samples ranged from 5.82 to 6.17 and similarly decreased to a final range of 3.98 to 4.19. In contrast, final titratable acidity varied among samples, with KSA exhibiting a significantly high value (1.09%) and CK the lowest value (0.95%). Notably, after two weeks of fermentation, the acidity of KSA reached 0.93%, whereas that of CK remained at 0.63%. This finding indicates a significantly faster rate of acid production in the KSA group than in the CK group. Overall, kimchi samples containing salted seafood exhibited faster and higher acidity values compared to the CK, suggesting that salted seafood accelerates fermentation. Previous studies have also indicated that kimchi supplemented with salted seafood exhibits a faster increase in acidity than those without it (Choi et al., 2018; No et al., 1995). Notably, the high nitrogen content in salted seafood promotes microbial growth and accelerates the fermentation process of kimchi (Park & Kim, 1991). Therefore, our results may be attributed to the elevated initial microbial load and nutrient availability provided by salted seafood, which promotes rapid lactic acid fermentation.

Throughout the fermentation period, TN and AN levels were consistently higher in all groups supplemented with salted seafood than in the CK group (Fig. 1A). Notably, the KSA group exhibited the highest TN and AN levels, with the AN levels being approximately 1.4 times higher than those in the CK group after 4 weeks of fermentation. This increase in AN is particularly relevant, as it enhances umami taste and reflects active proteolytic breakdown of fish proteins and peptides into free amino acids (Choi et al., 2018; Rhee et al., 2011). In our previous study, salted anchovy exhibited notably high levels of TN and AN, suggesting the abundance of proteolytic enzymes or microorganisms capable of utilizing them (Lim et al., 2024). In the present study, we confirmed that adding salted anchovies or other types of salted seafood to kimchi significantly affected the total protein content and its subsequent degradation during fermentation. Given the comparable salinity among treatments, the enhanced TN and AN levels are likely attributable to seafood-derived microbial and enzymatic activities that facilitate proteolysis during fermentation instead of osmotic stress. This finding suggests that salted seafood may release elevated levels of protein-related compounds and undergo efficient proteolysis in kimchi during fermentation.

#### 3.1.2 Changes in microbial characteristics

The microbial counts of the various kimchi samples prepared in this study are presented in Fig. 1B. Aerobic and lactic acid bacteria counts increased rapidly during the initial two weeks in all samples, ultimately reaching a stationary phase. This result is consistent with previous studies demonstrating a rapid increase in aerobic and lactic acid bacteria populations within the first 2–3 weeks of kimchi fermentation (Lee et al., 2022; Lim et al., 2022; Song et al., 2021). By week 6, coliform counts declined to below the detection limit in all groups. Notably, this reduction was observed earlier in the CK and KSA groups, occurring as early as week 3. Coliforms exhibit limited proliferation at pH levels below 4.5 (Knarreborg et al., 2002). Accordingly, the continuous increase in acidity during kimchi fermentation likely contributed to the reduction of pH, establishing conditions that inhibited coliform growth.

In our study, yeast and mold counts exhibited minor fluctuations and remained comparable among the salted seafood groups during the early fermentation period. However, after week 4, they exhibited a gradual increase in most groups, except for KSH, coinciding with a slight decline in LAB abundance. This temporal pattern is consistent with the microbial succession described by Jeong et al. (2013), who reported increases in yeast populations following the decline in lactic acid bacteria during the later stages of kimchi fermentation.

### 3.2 Microbial community composition

**Fig. 2:**
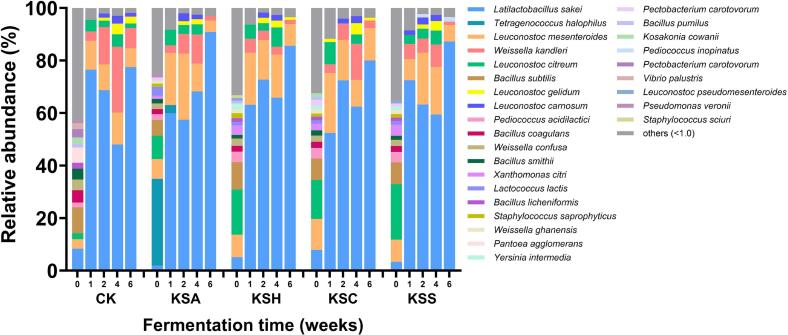
Bacterial community composition at the species level in kimchi prepared with different types of salted seafood. The composition was determined using the SILVA rRNA database. “Others” indicate a percentage of reads less than 1.0% of the total reads in all samples in analyses at species levels.

We analyzed the bacterial community composition of kimchi fermented with different types of salted seafood at the species level using the SILVA rRNA database, and the results are presented in Fig. 2. At week 0, the microbial composition of the CK was highly diverse, whereas salted seafood-supplemented samples were dominated by certain bacterial taxa. Specifically, in the KSA group, *Tetragenococcus halophilus* accounted for approximately 33.1% of the community. This finding is consistent with our previous study (Lim et al., 2024), in which *T. halophilus* was the predominant species throughout the fermentation of salted anchovy. Collectively, these findings suggest that *T. halophilus* was introduced into the kimchi via the added salted seafood, supporting the role of salted seafood as a microbial inoculant. In addition, *T. halophilus* is a species commonly present in high-salt fermented foods and exhibits proteolytic activity under saline conditions (Han, Wang, et al., 2024; Udomsil et al., 2011; Yang et al., 2023). Therefore, the elevated AN levels observed in the KSA group may be attributed to the activity of *T. halophilus.* Our findings also indicated that all salted seafood-supplemented groups commonly harbored *Leuconostoc citreum* from the beginning of fermentation. Notably, we also detected *Bacillus subtilis* across all groups, including the CK.

During the first week of fermentation, *Latilactobacillus sakei* rapidly became dominant in all samples, accounting for more than half of the total community, followed by *Leuconostoc mesenteroides*. This microbial profile remained generally stable through the later stages of fermentation, although the rate of change in species abundance varied slightly among groups. Notably, we observed a marked increase in the abundance of *Weissella kandleri* in the CK group at week 4, followed by a decrease, suggesting potential instability in fermentation dynamics in the absence of salted seafood. This observation supports the hypothesis that the addition of salted seafood contributes to microbial stabilization during fermentation. Overall, these results indicate that salted seafood introduces a diverse microbial community and functions as a natural microbial starter in fermentation, promoting both microbial consistency and fermentation stability.

### 3.3 Changes in nucleic acid-related compounds and free amino acid content during fermentation

#### 3.3.1 Nucleic acid-related compounds

**Fig. 3:**
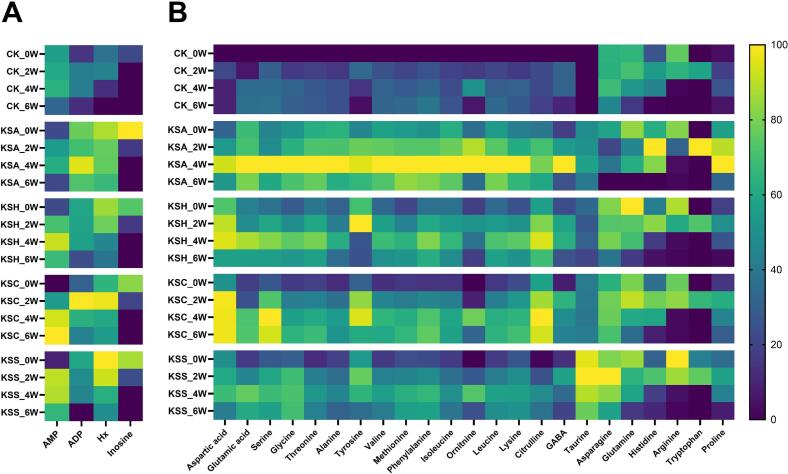
Heatmaps of (A) nucleic acid and (B) amino acid composition in kimchi during fermentation. The heatmaps represent the relative abundance of these two compounds throughout fermentation (0, 2, 4, and 6 weeks) and in different kimchi samples. Each column is normalized to represent the relative abundance of individual components across samples.

The degradation of adenosine triphosphate (ATP) and nucleic acid-related compounds in fish and shellfish muscle proceeds through a series of enzymatic reactions catalyzed by endogenous enzymes, occurring in the following order: adenosine diphosphate (ADP) → adenosine monophosphate (AMP) → inosine monophosphate (IMP) → inosine → hypoxanthine (Hx) → xanthine → uric acid (Fox, 1981). Nucleic acid-related compounds, such as AMP, ADP, inosine, and Hx, contribute to the umami and bitter taste in fermented foods (Fuke & Konosu, 1991). Therefore, these compounds may influence the sensory profile of kimchi. We analyzed nucleic acid-related compounds in kimchi samples, and the results are presented in Fig. 3A.

At the beginning of fermentation (0 W), the CK group exhibited relatively low levels of nucleotides compared to the salted seafood-supplemented kimchi samples. As fermentation progressed, ADP and AMP levels initially increased before subsequently declining in the CK group. However, in the KSC group, AMP levels continued to rise without a subsequent decrease, reaching the highest levels on week 6. Li et al. (2017) reported that ATP degradation in fish is more closely associated with endogenous enzymes derived from the fish itself than with microbial activity. Considering that microbial community compositions became increasingly similar among the salted seafood-treated groups as fermentation progressed (Fig. 2), the distinct nucleotide patterns observed are more likely attributable to species-specific differences in substrate-derived endogenous enzymatic activities rather than to microbial metabolism alone. In fish muscle, ATP degradation involves sequential reactions: adenylate kinase catalyzes the conversion of ADP to AMP (EC 2.7.4.3), which is subsequently deaminated to IMP by AMP deaminase (AMPD, EC 3.5.4.6). Therefore, yellow croaker muscle may contain relatively elevated adenylate kinase activity and/or comparatively decreased AMPD activity, which could contribute to sustained AMP accumulation in the KSC group during fermentation.

Throughout fermentation, Hx levels remained significantly higher in the salted seafood-supplemented groups than in the CK groups. In addition, all samples exhibited a decreasing trend in both compounds during fermentation. Although inosine levels were significantly higher in salted seafood-supplemented groups than in the CK group at early stages, they dropped below the detection limit in all samples after week 2. In our previous study on kimchi with different salted shrimp varieties, inosine was detected only at the early stage and completely depleted thereafter, whereas Hx increased throughout fermentation (Lim et al., 2022). Although Hx did not continuously increase during fermentation in the present study, the KSS group, which contained shrimp-based salted seafood, consistently exhibited relatively high Hx levels. This finding suggests that the use of crustacean-based salted seafood may lead to elevated Hx accumulation in kimchi compared to fish-based variants. Therefore, the choice of raw material species for salted seafood plays a critical role in shaping the flavor profile of kimchi.

#### 3.3.2 Free amino acids

In kimchi, amino acids such as glutamic acid, glycine, and alanine enhance umami taste and contribute to overall flavor quality (Kim & Kim, 2013). Therefore, we profiled the amino acids present in the kimchi samples, and the results are presented in Fig. 3B. The CK group exhibited the lowest concentrations of most amino acids, consistent with the low TN and AN levels, which are indicators of protein degradation. This finding suggests that the addition of salted seafood induces proteolytic activity, likely mediated by endogenous enzymes or microorganisms derived from the salted seafood. As fermentation progressed, the levels of most amino acids increased. However, glutamine, histidine, and arginine exhibited decreasing trends, possibly owing to microbial utilization as nitrogen or energy sources. Notably, at week 4, the KSA group exhibited the highest concentrations of several amino acids, including glutamic acid, serine, glycine, and γ-aminobutyric acid (GABA). GABA is a bioactive compound produced through the microbial decarboxylation of glutamic acid. Its accumulation may be associated with *Lactobacillus* species, which are recognized for their GABA-producing capabilities (Hou et al., 2024; Lyte, 2011). In our previous study on salted seafood, salted anchovies exhibited the highest concentrations of most amino acids (Lim et al., 2024). These findings suggest that the elevated amino acid levels observed in the KSA group likely originated from the salted anchovy.

Although free amino acids accumulated progressively in all groups during fermentation, including the control, the salted seafood-supplemented kimchi consistently demonstrated markedly higher levels than the control at equivalent fermentation time points. This differential accumulation suggests that salted seafood contributes substantially to the overall amino acid pool independent of fermentation-driven proteolysis. It may also influence specific amino acid metabolic pathways through enzymatic and microbial mechanisms. Furthermore, supplementation with specific types of salted seafood may promote the accumulation of amino acids associated with flavor and bioactivity, highlighting their importance in modulating the sensory and functional quality of kimchi.

### 3.4 Metabolic profiles of kimchi supplemented with different types of salted seafood

**Fig. 4:**
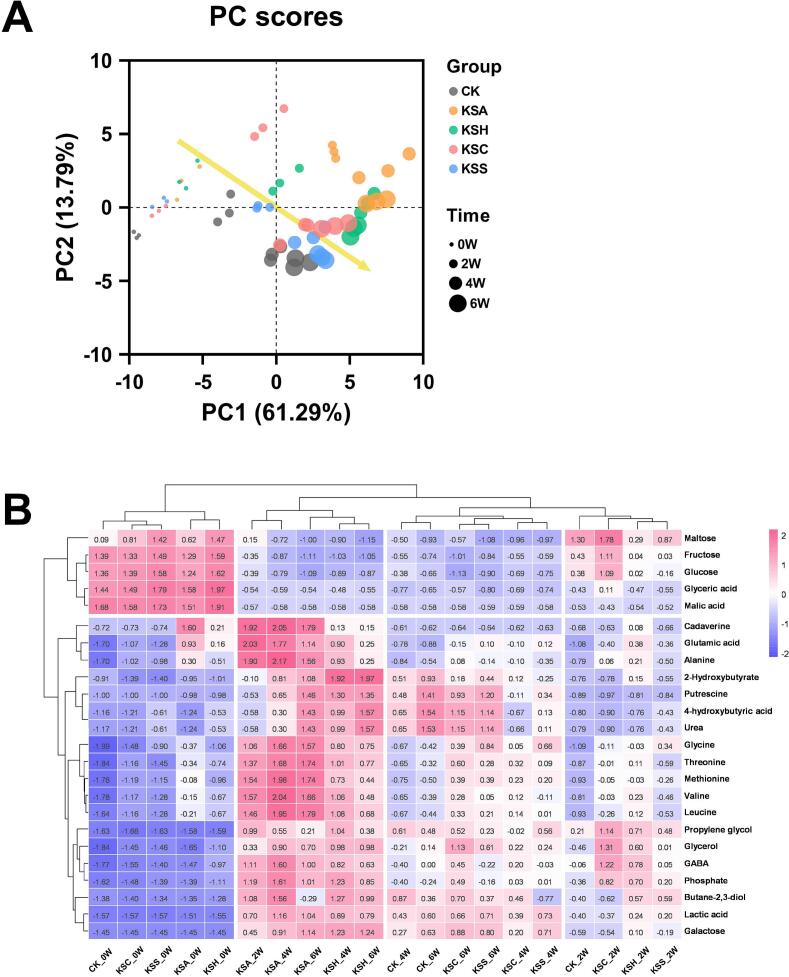
Principal component analysis (A) and heatmap (B) of the metabolic profiles in kimchi prepared with different types of salted seafood. Red and blue colors in the heatmap indicate higher and lower relative abundances, respectively. (For interpretation of the references to colour in this figure legend, the reader is referred to the web version of this article.)

PCA of the metabolite profiles (Fig. 4A) revealed clear differentiation among kimchi samples as a function of both fermentation time and salted seafood type. The first principal component (PC1), explaining 61.29% of the total variance, mainly reflected fermentation progression, as evidenced by the time-dependent rightward shift of all groups, indicating that the global metabolic landscape is primarily driven by fermentation time. In contrast, separation along PC2 captured differences among the different *jeotgal* treatments. Samples containing salted seafood were more widely dispersed along both PC1 and PC2, indicating greater metabolic plasticity, whereas CK samples formed a compact cluster, suggesting a relatively restricted metabolic trajectory in the absence of salted seafood. These patterns imply that *jeotgal* addition not only accelerates but also diversifies metabolic pathway utilization during kimchi fermentation.

The heatmap of the top 28 metabolites (Fig. 4B) further illustrates pathway-level changes in carbon and nitrogen metabolism. Sugars such as maltose, fructose, and glucose were abundant at 0–2 weeks and decreased thereafter, consistent with their consumption as primary carbon sources by fermentative microorganisms (Cho et al., 2006). In parallel, most organic acids and amino acids increased progressively during fermentation across all groups, reflecting activation of glycolytic and amino acid-releasing pathways. In contrast, malic acid production decreased during fermentation, likely reflecting the malolactic fermentation catalyzed by the dominant species L. *sakei*. This bacterium possesses malolactic enzyme activity and converts L-malate into L-lactate under fermentation conditions (Kolb et al., 1992; Oguro et al., 2017). Kimchi supplemented with salted seafood showed greater accumulation of glutamic acid, GABA, methionine, and valine than CK. These metabolites are associated with umami perception and bioactivity (Hou et al., 2024; Kim & Kim, 2013), indicating that *jeotgal* enhances both flavor-related and potentially functional metabolic output through intensified proteolysis and amino acid/γ-aminobutyric acid pathways. In the later fermentation stages, KSA, KSH, KSC, and KSS also exhibited elevated urea levels, which may result from increased protein inputs and subsequent deamination, followed by channeling of nitrogenous intermediates into the urea cycle.

Taken together, the combined PCA and heatmap analyses indicate that salted seafood substantially rewires metabolic fluxes in kimchi by accelerating sugar catabolism, stimulating amino acid and GABA production, and promoting the formation of flavor-active organic and nitrogenous compounds. Among the *jeotgal* types, salted anchovy (KSA) induced the most diverse and intense metabolite shifts, which is consistent with its higher nitrogen content and richer microbial load. We have previously demonstrated that salted anchovy itself undergoes the most dynamic metabolite remodeling during its own fermentation (Lim et al., 2024), and the present results confirm that this metabolic “activity” is transferred to the kimchi matrix when anchovy *jeotgal* is used. Importantly, by resolving *jeotgal* type–specific metabolic pathway signatures (e.g., malolactic conversion, GABA shunt, branched-chain amino acid accumulation, and urea cycle activation) in a single framework, the present study extends earlier reports that described only global increases in free amino acids or a limited set of organic acids in *jeotgal*-treated kimchi. This pathway-level view provides a chemically grounded explanation of how each salted seafood acts as a distinct metabolic driver of kimchi fermentation.

### 3.5 Sensory attribute profiles evaluated using an e-tongue

**Fig. 5:**
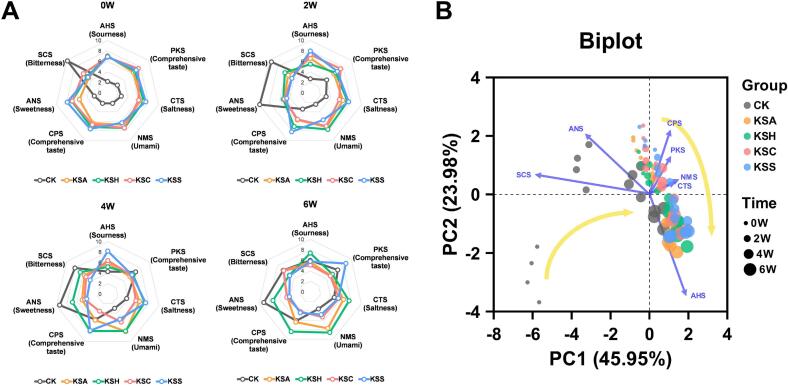
Sensory attribute profiles of kimchi samples evaluated using an electronic tongue (e-tongue). (A) Radar charts present the e-tongue response signal values. (B) Principal component analysis biplot depicts the distribution of kimchi samples based on sensory characteristics. Different colors indicate kimchi types, and dot sizes represent the fermentation periods (0, 2, 4, and 6 weeks). Arrows indicate the contribution of sensory attributes to the principal components.

The e-tongue is a sensor-based analytical device that simulates human taste perception by detecting and quantifying multiple taste modalities, including sourness, umami, bitterness, and saltiness (Lvova, 2016). Radar plots of e-tongue measurements (Fig. 5A) revealed temporal shifts in sensory characteristics across all kimchi samples in the present study. At week 0, most samples exhibited relatively low intensities across all taste modalities. However, the SCS value was significantly higher in the CK group than in the other groups. Notably, the CK group exhibited consistently lower responses across most sensors, including comprehensive taste indicators (PKS and CPS), than the salted seafood-supplemented groups. This finding suggests that the absolute amounts of proteins, amino acids, and other flavor-related compounds were relatively low in the absence of salted seafood. Consequently, the lack of dilution by other taste-contributing compounds may have amplified the perception of bitterness, leading to enhanced responses in the SCS sensor readings. In contrast, kimchi samples supplemented with salted seafood likely contained elevated initial levels of umami-related compounds, which may have partly diminished the detection of bitterness.

This finding suggests that the taste of CK may be relatively monotonous owing to its dominance of SCS, making it unlikely to exhibit a complex flavor. As fermentation progressed, there was a general increase in sourness (AHS) and umami (NMS), which are typical sensory indicators of lactic acid fermentation and amino acid accumulation in all groups. By week 6, all samples exhibited elevated responses in AHS and NMS, indicating fermentation. Among the samples, the KSA and KSH samples consistently displayed higher umami and comprehensive taste (PKS, CPS) scores than the CK, especially at weeks 4 and 6. This finding suggests that the addition of salted seafood enhances flavor complexity, likely owing to its elevated contents of amino acids and nucleotides. Consistent with our results, Lee et al. (2024) reported that the type of starter culture used in kimchi affected the results of electronic tongue analysis. Therefore, the microbial succession altered by the addition of salted seafood may have enhanced flavor complexity, further supporting its role as a natural starter culture in kimchi fermentation.

The PCA biplot depicted in Fig. 5B summarizes the sensory variability among samples. PC1 and PC2 accounted for 45.95% and 23.98% of the total variance, respectively. Samples were clustered distinctly based on fermentation time, with early-stage samples (0 W, smaller dots) positioned to the upper left and late-stage samples (6 W, larger dots) distributed along the lower right side of the plot. This pattern suggests progressive sensory development over time. In the CK group, the sensory profile at the early stage of fermentation was distinctly separated from the other samples, likely owing to its notably high bitterness (SCS) level. This finding suggests that the strong bitterness and low umami perception in the CK group may negatively affect the overall taste quality of kimchi. Therefore, the presence of salted seafood can lead to significant differences in sensory characteristics.

### 3.6 Correlation analysis among metabolites, microbial community, and sensory attributes

**Fig. 6:**
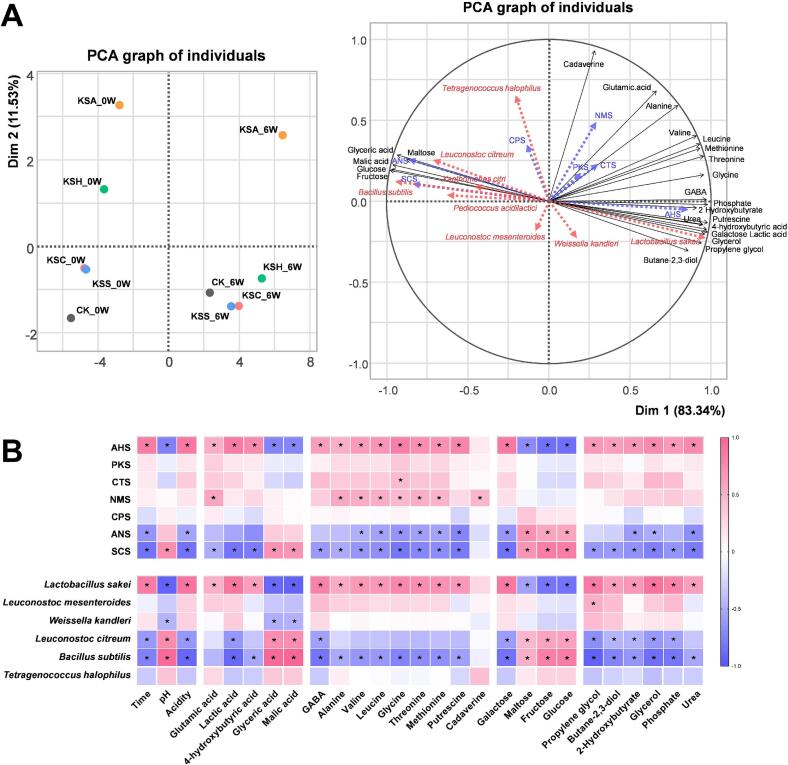
Principal component analysis (PCA) of metabolites and heatmap of Pearson's correlation coefficients. (A) Principal component analysis of kimchi samples based on metabolic data from weeks 0 and 6. Microbial genera (in red) and sensory attributes (in blue) are included as supplementary variables. (B) Heatmap of Pearson's correlation coefficients between sensory parameters or dominant microbial genera and physicochemical properties (metabolites). Red and blue indicate positive and negative correlations, respectively. Asterisks (*) denote statistically significant correlations (*P* < 0.05). (For interpretation of the references to colour in this figure legend, the reader is referred to the web version of this article.)

As presented in Fig. 6A, we performed PCA using metabolic profiles of samples collected at weeks 0 and 6 of fermentation, representing the start and end points of fermentation. We included microbial genera and e-tongue-based sensory attributes as additional variables. We observed a clear separation between samples collected on weeks 0 and 6 along PC1, explaining 83.34% of the variance. This finding suggests significant metabolic transformation throughout fermentation. Before fermentation, kimchi samples were strongly associated with sweetness (ANS) and bitterness (SCS), and microbial species such as L. *citreum*, *B. subtilis*, and *T. halophilus* appeared to play a key role in the metabolism of early-stage sugars such as glucose, fructose, and maltose. In contrast, post-fermentation samples exhibited strong associations with sourness (AHS) and umami (NMS), with L. *sakei* playing a major role in amino acid and organic acid metabolism.

The upper portion of the correlation heatmap presented in Fig. 6B illustrates the relationships between sensory attributes and compositional characteristics, while the lower portion depicts the correlations between major microbial species and the same characteristics. Sourness (AHS) exhibited positive correlations with fermentation time, acidity, and several metabolites, including glutamic acid, lactic acid, and other organic and amino acids, but negative correlations with pH, malic acid, and various sugars. In contrast, bitterness (SCS) and sweetness (ANS) demonstrated opposite correlation patterns to AHS. Similarly, *L. sakei* displayed a correlation profile consistent with AHS, whereas L. *citreum* and *B. subtilis* were more closely associated with SCS. These findings suggest that the metabolic activity of specific microbial taxa is directly linked to sensory characteristics.

Overall, microbial fermentation shifts the initially bitter flavor of unfermented kimchi into a more complex and desirable flavor profile characterized by sourness and umami. Moreover, the microorganisms that dominate this transformation vary depending on the type of salted seafood added, influencing both the fermentation rate and the concentration of specific metabolites and flavor compounds. Therefore, salted seafood serves as a seasoning agent while contributing endogenous enzymes and microbes that influence fermentation.

## 4 Conclusion

In the present study, we revealed that different types of salted seafood markedly modulate the microbial dynamics, metabolic pathways, and sensory attributes of kimchi during fermentation. Salted seafood accelerated acidification and proteolysis, leading to elevated levels of glutamic acid, GABA, and other flavor-active metabolites, with salted anchovy exerting the strongest effects, consistent with its higher nitrogen content and enzymatic/microbial activity. *Jeotgal* addition introduced specific early-stage taxa such as *T. halophilus* and L. *citreum*, which were subsequently replaced by L. *sakei*-dominated communities closely associated with umami-enhancing metabolites and organic acids, whereas kimchi without salted seafood exhibited delayed acid production, less stable microbial behavior, and a more bitter, less umami sensory profile. By integrating microbial profiling, metabolite analysis, and electronic tongue data, this work supports the notion that salted seafood functions not only as a flavoring ingredient but also as a natural microbial and enzymatic starter, providing a mechanistic basis for selecting *jeotgal* types to achieve targeted flavor profiles and improved product consistency.

These findings suggest that *jeotgal* selection can be strategically optimized to align with specific goals. For example, salted anchovies may be preferable when rapid acidification and strong umami development are desired. Thus, *jeotgal* could be regarded as a functional fermentation module that influences both fermentation kinetics and final flavor profile. Nevertheless, certain limitations should be acknowledged. In this study, enzyme activities were not directly quantified, and industrial-scale validation was not performed. Therefore, future studies integrating enzyme assays, functional metagenomics, and pilot-scale fermentation trials would further clarify species-specific mechanisms and support process optimization. Overall, this study advances our understanding of ingredient-driven fermentation ecology and provides a scientific basis for the rational selection of *jeotgal* to achieve targeted sensory characteristics and improved product consistency.

## CRediT authorship contribution statement

**Ju Young Lim:** Writing – original draft, Formal analysis, Data curation, Conceptualization. **Yun-Jeong Choi:** Validation, Formal analysis, Conceptualization. **Ji-Young Choi:** Visualization, Data curation, Conceptualization. **Sung Hee Park:** Methodology, Investigation, Conceptualization. **Sung Gi Min:** Project administration, Conceptualization. **Mi-Ai Lee:** Writing – review & editing, Supervision.

## Fundings

This research was supported by a grant from the 10.13039/501100003722World Institute of Kimchi (KES2603), funded by the Ministry of Science and ICT, Republic of Korea.

## Declaration of competing interest

The authors declare that they have no known competing financial interests or personal relationships that could have appeared to influence the work reported in this paper.

## Data Availability

Data will be made available on request.
